# Enhanced Cloud Detection Using a Unified Multimodal Data Fusion Approach in Remote Images

**DOI:** 10.3390/s25092684

**Published:** 2025-04-24

**Authors:** Yan Mo, Puhui Chen, Wanting Zhou, Wei Chen

**Affiliations:** 1 College of Aeronautics Engineering, Nanjing University of Aeronautics and Astronautics, Nanjing 210016, China; 2 School of Information Engineering, Nanchang Hangkong University, Nanchang 330063, China; 2304085401015@stu.nchu.edu.cn; 3 State Key Laboratory of Mechanics and Control of Mechanical Structures, Nanjing University of Aeronautics and Astronautics, Nanjing 210016, China; phchen@nuaa.edu.cn; 4 College of Geoscience and Surveying Engineering, China University of Mining & Technology, Beijing 100083, China; chenw@cumtb.edu.cn

**Keywords:** multimodal cloud detection, unified fusion approach, plug-and-play module, feature fusion strategy

## Abstract

Aiming at the complexity of network architecture design and the low computational efficiency caused by variations in the number of modalities in multimodal cloud detection tasks, this paper proposes an efficient and unified multimodal cloud detection model, M2Cloud, which can process any number of modal data. The core innovation of M2Cloud lies in its novel multimodal data fusion method. This method avoids architectural changes for new modalities, thereby significantly reducing incremental computing costs and enhancing overall efficiency. Furthermore, the designed multimodal data fusion module possesses strong generalization capabilities and can be seamlessly integrated into other network architectures in a plug-and-play manner, greatly enhancing the module’s practicality and flexibility. To address the challenge of unified multimodal feature extraction, we adopt two key strategies: (1) constructing feature extraction modules with shared but independent weights for each modality to preserve the inherent features of each modality; (2) utilizing cosine similarity to adaptively learn complementary features between different modalities, thereby reducing redundant information. Experimental results demonstrate that M2Cloud achieves or even surpasses the state-of-the-art (SOTA) performance on the public multimodal datasets WHUS2-CD and WHUS2-CD+, verifying its effectiveness in the unified multimodal cloud detection task. The research presented in this paper offers new insights and technical support for the field of multimodal data fusion and cloud detection, and holds significant theoretical and practical value.

## 1. Introduction

Cloud detection, as a core task within the realm of remote sensing technology, holds a pivotal position in numerous key areas such as weather prediction [1], climate simulation [2], and environmental monitoring [3]. With the swift advancements in science and technology, a diverse array of technical methodologies has emerged to enhance both the accuracy and efficiency of cloud detection processes.

Traditional methods are mainly based on threshold setting [4,5], statistical analysis [6,7] and physical modeling [8,9], which provide a solid foundation for an in-depth understanding of the cloud characteristics. These approaches reveal the basic properties and distribution rules of clouds through careful analysis of remote sensing data. While effective in many scenarios, they face significant limitations: threshold-based methods often fail to distinguish clouds from snow due to their nearly identical reflectance in visible and near-infrared bands; statistical approaches rely heavily on manual feature engineering; and physical models are constrained by predefined rules that cannot adapt to complex, dynamic cloud patterns. These fundamental constraints hinder their performance in challenging detection scenarios.

To address these limitations, deep learning has emerged as a powerful alternative, bringing revolutionary changes to cloud detection. In particular, Convolutional Neural Network (CNN) has become a mainstream tool in cloud detection tasks with its excellent feature extraction and classification capabilities, significantly improving the accuracy of detection [10,11,12]. Some scholars introduce branch networks as the auxiliary of the backbone network [13,14,15] to enhance the performance of feature extraction. At the same time, the introduction of Transformer architecture further enhances the robustness and generalization performance of cloud detection models with its powerful ability to capture long-distance dependencies and context information [16,17,18].

However, in complex and variable scenes, the feature similarity between clouds and ground highlighted background (especially snow and water area) is always a challenging problem in cloud detection. With the continuous progress of satellite technology, the acquisition of remote sensing data has become increasingly abundant [19]. Multimodal learning technology has opened up a new path for improving the performance of cloud detection through the efficient deep fusion of information. But the existing multimodal methods [20,21] remain fundamentally limited by their requirement for dedicated backbone networks per modality—an architecture that becomes increasingly inefficient as new modalities are introduced. In view of this, we face a challenging research issue: how to design and implement an efficient and unified multimodal framework that has the ability to process any number of modal data to construct a mode-independent cloud detection model.

**Figure 1:**
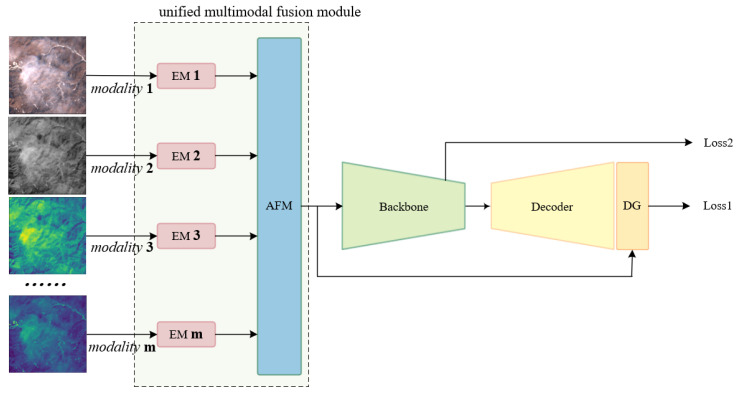
An overview of M2Cloud.

To address the aforementioned challenges, this paper introduces a highly scalable, unified multimodal cloud detection model designated as M2Cloud, which incorporates two key advances over conventional multimodal frameworks. First, our modality-agnostic fusion strategy eliminates the need for architectural modifications when incorporating new modalities. Second, the system demonstrates plug-and-play interoperability across backbone architectures. The architectural framework of M2Cloud is illustrated in Figure 1. The primary contributions of this study are delineated as follows:

- A novel approach to multimodal data fusion: When confronted with the task of processing a variable number of modalities, we reconsider the fusion strategy for multimodal data and devise a scheme that obviates the need for structural modifications to the network architecture with the introduction of each new modality. This innovative approach minimizes the incremental computational cost associated with each additional mode, thereby enhancing overall efficiency. Furthermore, the multimodal data fusion module possesses exceptional extrapolation capabilities and can be seamlessly integrated into other network architectures in a user-friendly, plug-and-play fashion. This characteristic augments the practicality and flexibility of the module.
- Construction and exploration of a multimodal cloud detection model:The proposed M2Cloud model demonstrates remarkable performance, achieving or even surpassing SOTA accuracy levels on public multimodal datasets through the deep integration of multimodal data. This outcome not only validates the efficacy of the M2Cloud model in unified multimodal cloud detection tasks but also offers a viable reference methodology for the construction of similar models.

## 2. Related Work

### 2.1. Cloud Detection Method Based on Deep Learning

Convolutional Neural Networks (CNNs) have exhibited exceptional capabilities in extracting spectral and spatial features, leveraging their local receptive fields, weight sharing, translation invariance, and pooling techniques. These attributes enable deep architectures to efficiently capture high-level semantic representations, which are pivotal for remote sensing cloud detection tasks [22]. However, the conventional encoder–decoder structure often encounters issues such as spatial information loss and feature dilution. To address these challenges, several studies [23,24,25] have incorporated attention mechanisms, including attention modules, into their frameworks. Furthermore, in an effort to bolster the capacity of deep learning networks to grasp intricate information characteristics, researchers have delved into multi-feature fusion strategies [15,26,27].

CNNs excel in local connectivity and feature extraction but struggle with capturing global context information. Conversely, Transformer models, exemplified by ViT (Vision Transformer) [28] and Swin Transformer [29], are adept at global feature extraction and contextual understanding. Singh introduced SSATR-CD, a vision transformer-based method for cloud detection in Sentinel-2 images [30]. Recognizing the strengths of Transformers in global feature extraction and CNNs in local feature extraction, several studies have integrated Transformers with CNNs to enhance the performance of cloud detection networks [31]. Gu et al. [32] employed a hybrid approach that combines Transformer and CNN models to concurrently extract semantic and spatial details from images, particularly when dealing with high- and low-resolution images. MAFNet [33] proposed a multi-branch attention fusion network that integrates ResNet50 dual-branch and Swin Transformer for feature extraction. The Multi-Branch Attention Fusion Module (MAFM) in MAFNet enhances location information through positional encoding and utilizes Multi-Branch Aggregation Attention (MAA) to fuse features of the same level from the two branches, thereby improving the capabilities for boundary segmentation and small object detection. These hybrid methods underscore the considerable potential of combining CNNs with Transformers for cloud detection tasks. But it is important to note that they primarily focus on feature-level fusion within single-modality data rather than addressing the challenges of multimodal fusion.

### 2.2. Multimodal Cloud Detection Method

Visible light is extensively utilized in satellite imagery and various other domains owing to its high definition and abundant informational content. Cloud identification primarily involves the analysis of grayscale values within the red, green, and blue channels, or the examination of the image’s texture and structure. Notably, clouds exhibit a heightened reflectance in the near-infrared band (NIR), resulting in a pronounced contrast against the background, thereby facilitating their identification and differentiation [34]. The integration of multi-spectral data from both visible and near-infrared light constitutes a pivotal approach in multimodal cloud detection methodologies [35,36,37]. This strategy markedly enhances the accuracy and robustness of cloud detection processes.

**Figure 2:**
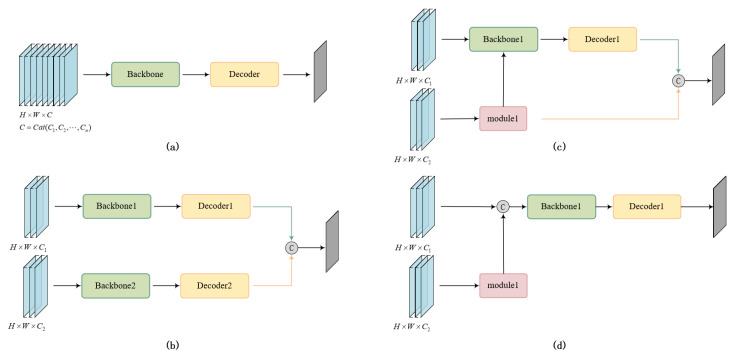
Multimodal fusion methods in existing cloud detection networks. (**a**) Simple concatenation of multimodal data along the channel dimension. (**b**–**d**) Specialized dual-modal fusion architecture.

Effectively distinguishing clouds from high-brightness backgrounds (such as snow, water bodies, etc.) in complex scenarios solely based on visible and near-infrared (VNIR) bands still poses significant challenges [38,39]. Mid- and short-wavelength infrared (SWIR) channels can be utilized to differentiate clouds from bright backgrounds due to their unique spectral response characteristics [40]. In particular, SWIR bands exhibit notable advantages in distinguishing clouds from snow-covered areas [41,42]. Meanwhile, vegetation, water bodies, urban areas, barren lands, and other land covers exhibit significant differences in top-of-atmosphere (TOA) reflectance across different spectral bands compared to clouds, providing abundant feature information for land cover classification [43]. Geographic information offers important prior information for cloud and snow detection in remote sensing images [21]. The integration of geospatial data enhances the spatial localization capability of models used for cloud detection [44,45]. However, when processing multimodal data, these methods often adopt a strategy of simply concatenating images from different modalities as input to the feature extraction network as illustrated in Figure 2a. This approach overlooks the complex correlations and complementarity that exist among multimodal data across different bands and types, thereby limiting the depth and breadth of information utilization from multimodal data.

Wu et al. [21] specifically designed a “geographic information encoder” that encodes the elevation, latitude, and longitude of the image into a set of auxiliary maps, which are then input into the detection network. Ma et al. [46] fused the differential features of the image and top-of-atmosphere (TOA) reflectance information, reducing the misclassification of thin clouds and bright surface features, as well as missed detections. Fan et al. [47] guided the model to focus on the spatial relationships between clouds and cloud shadows in the context by fusing prior knowledge extracted from dark channels, thermal channels, and NDVI (Normalized Difference Vegetation Index). Xu et al. [48] proposed a novel gradient-aware feature aggregation module (GAFAM), which focuses on gradient changes within cloud boundary regions and utilizes gradient information to guide feature aggregation. These methods are limited to handling dual-modal situations and cannot effectively integrate more than two modal data. Furthermore, they typically employ separate feature extraction networks for each modality as illustrated in Figure 2b–d. This results in the need for complex redesign and adjustment of the original model whenever the number of modalities increases, significantly reducing efficiency.

### 2.3. Multimodal Datasets for Cloud Detection

The WHUS2-CD dataset [43], compiled by Wuhan University, includes Sentinel-2A imagery with 13 spectral bands at resolutions of 10 m, 20 m, and 60 m. It comprises 32 images, featuring diverse land covers like farmland, forests, and snow/ice. The dataset spans April 2018 to May 2020, covering all seasons. WHUS2-CD+ adds four images with snow/ice scenes.

The CloudSEN12+ dataset [49] expands CloudSEN12 [50], doubling expert-labeled annotations to become the largest for cloud and cloud shadow detection in Sentinel-2 imagery. It includes over 50,000 image patches with diverse cloud scenarios, labeled in two sizes: 509 × 509 and 2000 × 2000 pixels at 10 m resolution.

The GF1_WHU dataSets [51] cloud detection dataset, released by the SENDIMAGE Laboratory of Wuhan University, includes 108 GF-1 wide field of view (WFV) Level-2A scenes, along with corresponding reference cloud and cloud shadow masks. The dataset has a spatial resolution of 16 m and covers four key multispectral bands: red, green, blue, and near-infrared.

The MODIS dataset [40] is a fully annotated cloud detection dataset, segmented into 1192 training images, 80 validation images, and 150 test images. It is based on data from the Moderate Resolution Imaging Spectroradiometer (MODIS), one of NASA’s key Earth observation systems. The dataset includes the 10 most differentiated channels and covers a wide range of four main scenarios: ocean, land, sea–land interface, and polar glaciers.

The 38-Cloud dataset [52] has 18 training and 20 test Landsat 8 scenes. The 95-Cloud dataset [53], an extension, includes 75 diverse training scenes but uses the same 20 test scenes as the 38-Cloud. Both datasets feature red, green, blue, and near-infrared spectral channels.

The SPARCS dataset [54] covers 80 complete satellite scenarios derived from Landsat 8 satellites. In data processing, only bands 2 (blue), 3 (green), 4 (red), and 5 (near infrared) are selected as input channels.

Levir_CS dataset [21] is a large-scale dataset for cloud and snow detection, consisting of 4168 GF-1 WFV satellite images. These images exhibit global distribution characteristics, covering a variety of feature types such as plains, plateaus, water bodies, deserts, snow and ice, as well as combinations of these types. For each scene in the Levir_CS dataset, a four-band multispectral image, a digital elevation model (DEM) image, and a ground-truth annotated image are provided.

The specific information of these datasets above is shown in Table 1.

## 3. Method

### 3.1. Overall Network and Motivation

This paper endeavors to propose a unified multimodal cloud detection model capable of addressing quantity-independent modal tasks. To address the challenges associated with unified multimodal feature extraction, this paper employs two strategies:

1. The establishment of a shared, yet consistent feature extraction module for each modality, albeit with independent weights. This module is designed to learn the specific distribution characteristics within each modality while preventing the direct blending of modalities with substantial discrepancies, thereby preserving their inherent features. Additionally, a unique identifier, in the form of an inductive bias, is assigned to each modal category.
2. The utilization of cosine similarity enables the network to adaptively learn complementary features across different modalities, mitigating the redundancy of similar features.

**Figure 3:**
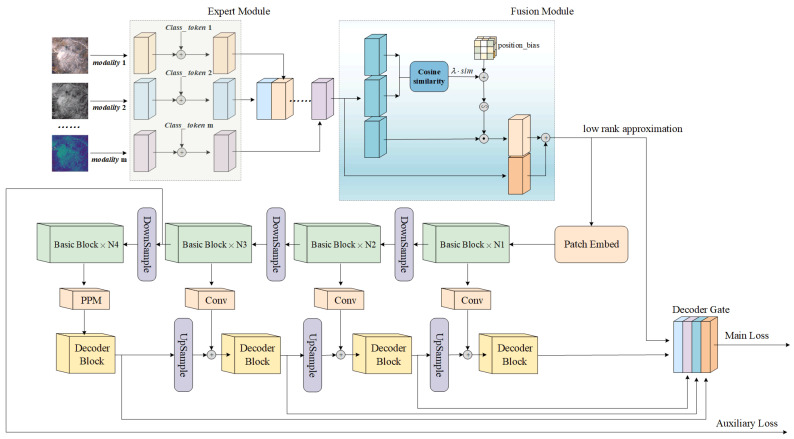
An overview of our proposed network. Unified Multimodal Cloud Detection Process: Data from various modalities are inputted into expert modules that share the same structure but possess independent weights. The extracted feature maps are then superimposed along the channel dimension. Complementary information is effectively fused by calculating cosine similarity. Subsequently, a low-rank approximation of the fused feature maps is fed into the backbone network for further feature extraction. Simultaneously, this approximation serves as an auxiliary input to the decoding gate, aiding in the decoding process of cloud detection.

Consequently, our model comprises four core modules: the Modal Feature Expert Module, the Adaptive Multimodal Fusion Module, the backbone network, and the decoder as illustrated in Figure 3. In the subsequent subsections, we delve into the specifics of each module.

### 3.2. Modal Feature Expert Module

In constructing a unified multimodal cloud detection model, it is vital to ensure that feature extraction modules across different modalities share a consistent structure, while each module maintains independent weights to capture modal-specific distribution characteristics. To avoid feature corruption from directly mixing disparate modalities, we adopt an inductive bias approach by assigning a unique identifier to each modality category. Specifically, we initialize these modal class labels using a truncated cosine function within the interval [α,β]. In practice, we employ a partial discrete sampling of cosine function values within [0,π].

Let $n_{\mathrm{branch}}$ be the number of modal branches and *i* be the branch index (from 0 to $n_{\mathrm{branch}}-1$), then inter-class identifier ${\mathrm{pos}}_{i}$ of the *i*th modality can be expressed as

(1) $${\mathrm{pos}}_{i}=\cos\left(\theta_{i}^{\mathrm{rad}}\right)$$

where $\theta_{i}^{\mathrm{rad}}$ represents the angle in radians calculated by

(2) $$\theta_{i}^{\mathrm{rad}}=\frac{135}{n_{\mathrm{branch}}}\cdot i\cdot \frac{\pi }{180^{\circ }}$$

Given the monotonically decreasing nature of the cosine function within the specified range, and by disrupting its inherent symmetry (considering only a portion of one half-period), each modal category can be assigned a unique, non-repeating cosine value as an identifier. This approach guarantees that, despite the potential similarities in features between two modal categories, their identifiers remain distinguishable due to different angles, thereby effectively mitigating the risk of multimodal features mutually canceling out in specific scenarios.

Furthermore, we associate gradient attributes with modality class labels and utilize matrix broadcast techniques to sample these identifiers, aligning them with the scale of the feature maps. This facilitates the network’s learning of token-level feature information during training. Consequently, we not only effectively leverage the complementary information among different modalities but also ensure the model’s stability in complex and dynamic environments. Notably, the model is capable of automatically discarding the modal class token in unimodal scenarios.

**Figure 4:**
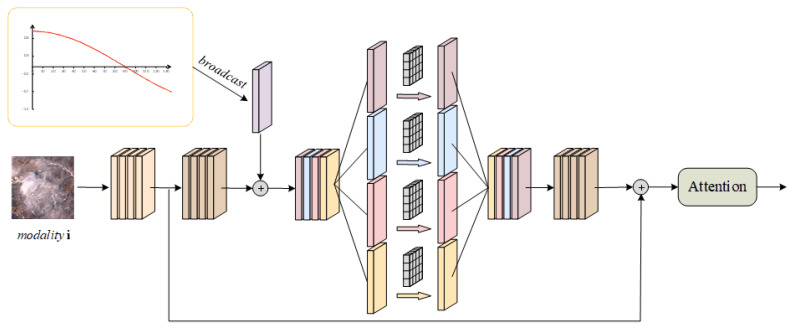
The architecture of the expert module.

Given the linear growth of computational complexity with the number of modes, lightweight design methodologies are integrated in this section. Specifically, each modal signal is input into the corresponding EM (expert module) processing unit, denoted as the i-th modality. As depicted in Figure 4, the initial features are mapped into a higher-dimensional space. To strike a balance between the feature extraction capability and computational efficiency, group convolution with a group number G is utilized, reducing the computational cost, model parameters, and floating-point operations (FLOPs) to 1/G of those required by standard convolution. Furthermore, residual connections are introduced to facilitate cross-layer feature propagation and fusion.

### 3.3. Adaptive Multimodal Fusion Module

The repeated stacking of similarity features fails to introduce novel perceptual information into the neural network. When dealing with multimodal data, the principal challenge lies in exploring and extracting complementary features among different modalities, while simultaneously preserving the semantic information of each modality in a compact, low-dimensional representation. This represents the core issue in achieving quantity-agnostic modal feature fusion.

From the perspective of spatial cosine distance, a larger cosine distance between two basis vectors (or feature vectors) indicates a more significant difference in their spatial orientation, often perceived as a potential indicator of feature complementarity. When two feature vectors from different modalities exhibit a large cosine distance in space, they may represent unique information or attributes specific to their respective modalities. In the fusion process, these features can complement one another, thereby providing a more comprehensive and accurate description of the data.

**Figure 5:**
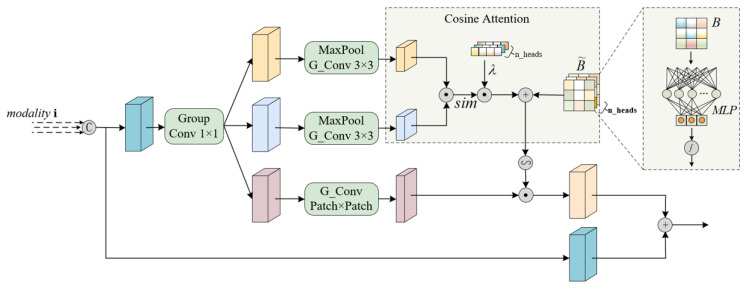
The architecture of the adaptive fusion module.

As shown in Figure 5, we denote the high-order multimodal matrix received by the EM as $X=[X_{1};X_{2};\ldots ;X_{n}]$, where the stacking of the n modalities is arranged solely along the channel dimension without fusion. Each modality can be decomposed into m individual subspaces in the channel dimension. Initially, X is replicated into three distinct copies through linear transformations, with each modality having a unique set of weights, represented as

(3) $${\tilde{Y}}_{1},{\tilde{Y}}_{2},\tilde{Z}=X\left[W_{1},W_{2},\ldots ,W_{n}\right]$$

where $len(W_{i})=3m$, for all i=1,2,…,n. For ${\tilde{Y}}_{1}$ and ${\tilde{Y}}_{2}$, the cosine similarity can be calculated using the following formula:

(4) $$S=Sim\left({\tilde{Y}}_{1},{\tilde{Y}}_{2}\right)=\lambda \cdot \frac{{\tilde{Y}}_{1}⊙{\tilde{Y}}_{2}}{∥{\tilde{Y}}_{1}{∥}_{2}{∥{\tilde{Y}}_{2}∥}_{2}}+\tilde{B}$$

where ⊙ denotes the Hadamard product. λ is a learnable modifier that scales the similarity scores to help stabilize the training process and improve the performance of the model. The initial value is log(10). During training, λ is restricted to between log(10) and log(100) by the formula

(5) $$\lambda_{\mathrm{new}}=\exp\left(\min\left(\log(\lambda ),\log(100)\right)\right)$$

to update. This means that the value of λ is cropped to no more than 100, thus avoiding the negative impact of too large or too small values on training. $\tilde{B}$ is added to the similarity score to take into account the relative position between elements. The position bias is processed by a multi-layer perceptron (MLP) and scaled by a sigmoid activation function. The specific formula is

(6) $$\tilde{B}=Sigmod\left(MLP\left(\mathrm{sgn}\left(B\right)\times \frac{\log_{2}\left(|B|+1\right)}{\log_{2}\left(8\right)}\right)\right)$$

(7) $$\mathrm{sgn}\left(B\right)=\left\{\begin{array}{c} 1,\;\;\;\;\mathrm{if}\;B>0\\ 0,\;\;\;\;\mathrm{if}\;B=0\\ -1,\;\;\mathrm{if}\;B<0 \end{array}\right.$$

where sgn(B) is the symbolic function of the position deviation *B*, which maps the size of the position deviation to a suitable range through the scaling factor.

After obtaining the cosine similarity matrix, the attention weights are calculated using the softmax function. Furthermore, the cosine distance matrix is derived and integrated with a copy of the data $\tilde{Z}$. This integration aims to allocate heterogeneous relationships. The fusion process follows the mathematical expression below:

(8) $$\hat{X}={\mathrm{Softmax}}_{2}\left(1-{\mathrm{Softmax}}_{1}\left(S\right)⊙\tilde{Z}\right)$$

Here, ${\mathrm{Softmax}}_{1}$ and ${\mathrm{Softmax}}_{2}$ denote the Softmax function applied in different dimensions, respectively. Here, the similarity is converted to the difference value, which is normalized and dot-multiplied with $\tilde{Z}$ to obtain the fused complementary feature representation $\hat{X}$.

To deeply fuse feature representations and effectively reduce dimensionality, we employ a global convolution operation that focuses on channel paths, extracts cross-modal shared features, and eliminates redundant information. Additionally, we introduce residual connections to maintain feature integrity and prevent performance degradation. The final features are

(9) $$G\left(X\right)=X+\hat{X}$$

### 3.4. Backbone

We previously highlighted the potential of combining CNNs and Transformers for cloud detection. However, existing hybrid models often adopt a parallel design, using CNN and Transformer modules independently as feature encoders. While this leverages the strengths of both, it increases model complexity and computational demands. Moreover, due to the inherent differences in feature extraction between CNNs and Transformers, current studies are limited to simple fusion strategies like concatenation or weighted summation, which may not fully capture the complex interactions between these features.

To address these issues, this paper proposes an innovative hybrid basic block that integrates CNNs and Transformers as the core component of the network backbone. Additionally, we design corresponding stacking principles to ensure flexible scalability, allowing the model to adapt to varying scales and complexity requirements.

#### 3.4.1. Basic Block

Multimodal feature maps encompass richer information dimensions, suggesting that when performing multimodal extrapolation, we may need to rely on a more extensive network architecture to accommodate diverse feature bases and comprehensively extract features. Within the basic block, we innovatively design two operator series structures: the Global Self-Attention Unit (G-SAU) and the Multigroup Depthwise Separable Convolution (M-DSC).

**Figure 6:**
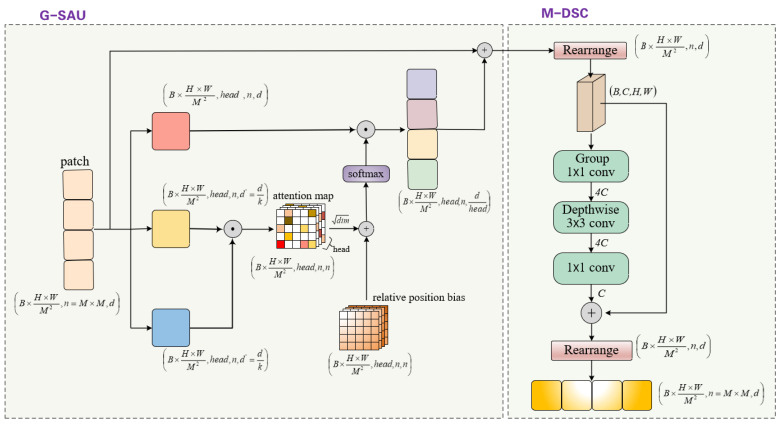
Structure of the basic block.

G-SAU, with its unconstrained perceptual dimension, excels in modeling long-distance dependencies. Meanwhile, CNN demonstrates high sensitivity to the contextual relationships between local pixels, aiding in compensating for the potential limitations of the self-attention mechanism in capturing local information. In M-DSC, we ingeniously adopt a convolution-based inverted residual structure to replace the feed-forward network comprising MLP in the conventional ViT. This design strategy facilitates the extraction of texture details from small target objects and further enhances the model’s feature extraction capability in complex multimodal scenes. The entire process can be referenced in Figure 6.

**In G-SAU**, despite the variations among different modal information, their spatial geometric information remains consistent. Therefore, multimodal self-attention is employed to learn these rich spatial features. The input feature map $X\in R^{C\times H\times W}$ is divided along the spatial dimensions into $\left(HW/M^{2}\right)$ non-overlapping patches of size n=M×M. Subsequently, different linear transformations are applied to the features of each patch, mapping them into three latent state spaces. To alleviate the computational burden, the dimensions of the queries (*Q*) and keys (*K*) are sparsified to 1/k of the original dimensions, while the dimensions of the values (*V*) remain unchanged. Within each window, for a feature matrix X composed of n tokens, the computation of self-attention is conducted as follows:

(10) $$S-\mathrm{Attention}=\mathrm{softmax}\left(\frac{\left(Q^{\prime }\cdot {\left(K^{\prime }\right)}^{T}\right)W^{l}}{\sqrt{d}}+B\right)\cdot VW^{n}$$

where $Q^{\prime }\in R^{\frac{n}{k}\times d^{\prime }},K^{\prime }\in R^{\frac{n}{k}\times d^{\prime }},V\in R^{n\times d}$. *d* and $d^{\prime }$ are the original and sparsified feature dimensions, respectively. $W^{l}$ and $W^{n}$ denote the light weights and normal weights, respectively, and *B* represents the relative position deviation matrix.

**In M-DSC**, we initially reshape the one-dimensional sequence features to revert them back into $X^{\prime }\in R^{C\times H\times W}$, thereby restoring their spatial structure:

(11) Reshape(X)=(bnd→BCHW)

(12) $$b=B\times \frac{H\times W}{M^{2}},d=C,n=M^{2}$$

To enhance feature representation, we introduce grouped pointwise convolutions for blocks with odd indices, dividing feature maps into g groups and applying independent linear transformations. For even-indexed blocks, we use standard convolutions. Within each group, depthwise separable convolutions (combining depthwise and pointwise convolutions) are employed. This diversifies feature transformations and boosts expressiveness. Group feature fusion is performed via a weighted sum along the channel dimension, constructing the final local perception representation:

(13) $$\hat{X}=X^{\prime }+{\mathrm{DS}}_{3\times 3}\left(\mathrm{ReLU}\left({\mathrm{Conv}}_{1\times 1}\left(X^{\prime }\right)\right)\right)$$

where $X^{\prime }$ and $\hat{X}$ represent the results after the self-attention operation and the output of the local perception unit, respectively; ${\mathrm{Conv}}_{k\times k}$ represents *k* × *k* (*k* = 1 here) convolution; and ${\mathrm{DS}}_{3\times 3}$ represents *k* × *k* (*k* = 3) depthwise separable convolution (depthwise convolution + pointwise convolution). With the aforementioned operations, we can efficiently model local relationships.

#### 3.4.2. Architecture Deployment

To construct a robust network architecture for parsing multi-scale semantic features, we devise a hierarchical, multi-resolution backbone. This backbone divides the feature extraction process into four distinct resolution levels, with each subsequent level being downsampled by a factor of two to capture a spectrum of information ranging from fine to coarse details.

In implementation, each resolution level comprises a predetermined number of identical basic blocks. We employ a differentiated configuration strategy: at the first, second, and fourth resolution levels (denoted as *N*1, *N*2, and *N*4, respectively, with *N*1 = *N*2 = *N*4 = 2), we maintain an equal number of basic building blocks to ensure stable and efficient feature extraction. Conversely, at the third resolution level, which may necessitate greater processing power due to the potential richness of semantic information, we augment the number of basic blocks (denoted as N3, with *N*3 = 16) to bolster the feature extraction capabilities.

### 3.5. Decoder

In our network architecture, we adopt the standard upsampling technique and skip connections as the core components of the decoder. At the lowest level of the network, we integrate the Pyramid Pooling Module (PPM) [55] to enhance the capability of capturing global context information.

**Figure 7:**
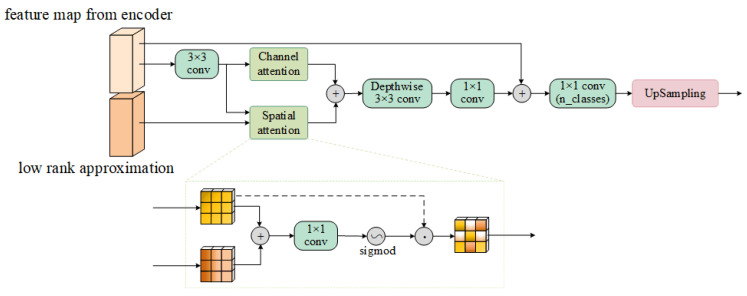
Structure of the decoder gate (DG).

To further improve the accuracy and efficiency of deep spatial indexing, we introduce the decoder gate (DG) mechanism, which represents an efficient second-order operation strategy. It is noteworthy that deep feature maps exhibit limitations in accurately capturing spatial details, and the DG mechanism ingeniously incorporates shallow feature maps as guidance. These shallow feature maps preserve finer spatial structures and edge details of the image, thereby effectively compensating for the blurriness of deep features in detailed expression. The structure of the proposed feature decoder gate is depicted in Figure 7. The process before accessing the classifier can be approximated as

(14) $$X_{\mathrm{out}}=X_{\mathrm{de}}+{\mathrm{DS}}_{3\times 3}\left(A_{\mathrm{channel}}\left(X_{\mathrm{de}}\right)+A_{\mathrm{spatial}}\left(X_{\mathrm{de}},X_{\mathrm{lra}}\right)\right)$$

where $X_{\mathrm{de}}$ and $X_{\mathrm{lra}}$ represent the feature map of the cascade at the decoder and the output of the approximate feature extraction module at the multimodal fusion module, and $A_{\mathrm{channel}}$ and $A_{\mathrm{spatial}}$ denote channel attention and spatial attention, respectively.

### 3.6. Loss Function

To solve the problem of feature degradation in deep networks, an auxiliary classifier is introduced into the third stage of the backbone network, which enhances feature representation by incorporating additional supervised signals to accelerate and optimize the learning process during the initial training stage. However, over-reliance on shallow auxiliary branches may weaken deep feature utilization and affect model performance. To this end, we design a dynamic loss allocation strategy. During the initial training stages, the auxiliary classifier is assigned a high loss weight (α) to provide strong supervisory signals that combat vanishing gradients and accelerate convergence, particularly crucial for capturing the spectral contrasts characteristic of cloud detection tasks. As training progresses, a linear decay factor ((α − β)/epochs) facilitates a smooth transition from auxiliary-to-main classifier dominance, maintaining stable optimization while allowing gradual adaptation to deeper feature representations. In the final training stages, a minimal auxiliary weight (β≈0.1α) preserves subtle supervisory signals without over-reliance on shallow features, ensuring the model develops robust deep feature discriminability while retaining sensitivity to fine cloud boundaries. The dynamic balance between auxiliary and main loss components effectively optimizes feature learning across all network depths while maintaining the model’s ability to capture both global cloud characteristics and local boundary details. The total loss can be expressed as follows:

(15) $$L_{\mathrm{total}}=L_{\mathrm{main}}+L_{\mathrm{aux}}\cdot \left(\alpha +\frac{\beta -\alpha }{\mathrm{epochs}}\cdot \mathrm{epoch}\right)$$

α and β (α>β) are two hyperparameters that denote the proportions allocated to the auxiliary branch at the beginning and end of the training, and $L_{\mathrm{main}}$ and $L_{\mathrm{aux}}$ denote the losses of the main and auxiliary branches, respectively.

## 4. Experiment

### 4.1. Datasets and Evaluation Metrics

In this study, the WHUS2-CD cloud detection special dataset constructed by Wuhan University is selected. Its core advantage is that it completely contains multi-spectral and multi-resolution information of 13 spectral bands of Sentinel-2 satellite, and provides complex surface scenes (farmland, forest, snow and ice, etc.) through 32 images covering the whole season. Especially for the technical difficulty of cloud–snow confusion, the enhanced version of WHUS2-CD+ specially adds four high-resolution images of snow and ice scenes, which strengthens the detection challenge of spectral overlap region. This dataset has been widely used as a benchmark library in the field of cloud detection. Its multi-spectral characteristics, seasonal coverage integrity and targeted enhancement design can effectively verify the robustness of the algorithm under different land surface conditions and meteorological scenarios, and fully support the technical verification requirements of the advantages of this method.

Since the spatial resolutions of the VNIR, VRE/SWIR, and Ca/WV/Cir bands are 10 m, 20 m, and 60 m, respectively, the WHUS2-CD dataset categorizes the 13 bands into three resolution-based groups of modals. Given that clouds exhibit higher reflectance in the near-infrared band (NIR), thereby creating a more significant contrast with the background, which aids in their identification and discrimination [34], the Near NIR band is treated as a separate model in this paper. To establish a unified multimodal processing framework, bilinear interpolation is employed to standardize the resolution of all bands to 10 m (including those originally at 20 m and 60 m).

The performance evaluation employs the conventional set of metrics widely adopted in cloud detection research (accuracy, precision, recall, F1-score, and MIoU), ensuring direct comparability with existing studies. Here are the definitions of the key metrics:

(16) $$\mathrm{Accuracy}=\frac{\mathrm{TP}+\mathrm{TN}}{\mathrm{TP}+\mathrm{TN}+\mathrm{FP}+\mathrm{FN}}$$

(17) $$\mathrm{Precision}=\frac{\mathrm{TP}}{\mathrm{TP}+\mathrm{FP}}$$

(18) $$\mathrm{Recall}=\frac{\mathrm{TP}}{\mathrm{TP}+\mathrm{FN}}$$

(19) $$F1\mathrm{-score}=2\cdot \frac{\mathrm{Precision}\cdot \mathrm{Recall}}{\mathrm{Precision}+\mathrm{Recall}}$$

(20) $$\mathrm{MIoU}=\frac{1}{N}\sum_{i=1}^{N}\frac{{\mathrm{TP}}_{i}}{{\mathrm{TP}}_{i}+{\mathrm{FP}}_{i}+{\mathrm{FN}}_{i}}$$

where TP, TN, FP, and FN stand for true positive, true negative, false positive and false negative, respectively. *N* is the number of classes in the dataset.

### 4.2. Implementation Details

Our PyTorch 3.9 implementation on an RTX 3090 GPU employs AdamW optimization ($\beta_{1}=0.9$, $\beta_{2}=0.999$) with 0.01 weight decay, using a linear warmup (5 epochs) to reach the initial learning rate of $2.0\times 10^{-4}$ followed by cosine decay over 100 epochs (minimum lr $1.0\times 10^{-6}$). We use cross-entropy loss with label smoothing (ϵ=0.1) and a batch size of 16 (due to memory constraints), along with random horizontal flipping (*p* = 0.5) and rotation ($\pm 15^{\circ }$) for data augmentation. These hyperparameters are determined through grid search on the validation set.

### 4.3. Main Properties

**Contribution of Multimodal Data to Network Performance**. To deeply analyze the specific impact of multimodal data on network performance, we design a benchmark model, Cloud_3, which utilizes only three visible optical bands (blue, green, and red) as the standard input for the single-modal cloud detection network. The Cloud_4 model builds upon this by adding the Near InfraRed band, thus using the complete VNIR (visible and near-infrared) band as input. The Cloud_10 model further incorporates six VRE/SWIR bands, while the Cloud_13 model adds three Ca/WV/Cir bands, encompassing all band data as input. Here, multiple bands are concatenated in the channel direction without any fusion, which constitutes the data input mode of most current multimodal cloud detection networks.

Experimental results on the WHUS2-CD dataset demonstrate significant performance differences among the three models as outlined in Table 2.

**Figure 8:**
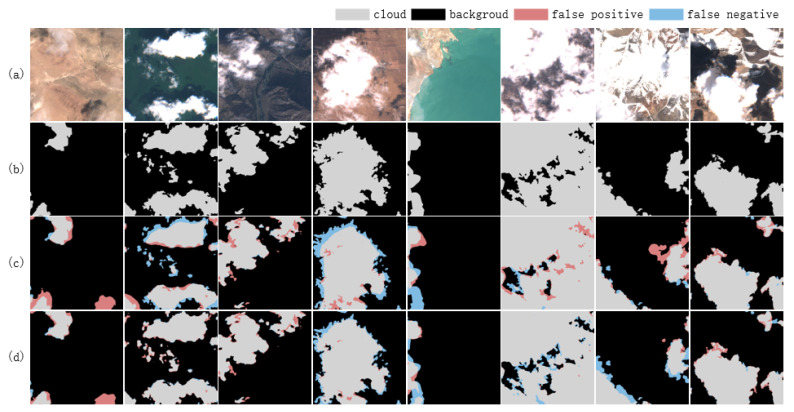
The important role of NIR band in cloud detection. (**a**) Image, (**b**) ground truth, (**c**) Cloud_3, and (**d**) Cloud_4.

As the input data modalities transition from three visible bands to thirteen multiband configurations, the cloud detection model demonstrates a progressive enhancement in its performance metrics, including accuracy, precision, recall, F1 score, and MIoU. A significant performance improvement is observed with the incorporation of the NIR band in the transition from Cloud_3 to Cloud_4, highlighting the critical role of the NIR band in cloud detection tasks as shown in Figure 8. Further enhancement is achieved with the addition of six VRE/SWIR bands in the transition from Cloud_4 to Cloud_10, although the improvement is less pronounced. In contrast, the transition from Cloud_10 to Cloud_13, which incorporates three Ca/WV/Cir bands, yields only marginal performance gains. Despite this, the Cloud_13 model achieves the highest values across all evaluation metrics, suggesting that incremental additions to the number of bands can sustainably enhance network performance, albeit with diminishing marginal returns. Furthermore, the recall rate exhibits a gradual improvement, indicating that the integration of multimodal data effectively reduces missed detections.

**Contribution of multimodal fusion to network performance.** To deeply investigate the specific impact of multimodal fusion on network performance, this study introduces an innovative Cloud_(3,1) model architecture. The Cloud_(3,1) model leverages the VNIR band; however, it innovatively treats visible light and NIR as distinct modal inputs. This approach is designed to deeply explore the complementarity among different modalities. Furthermore, the Cloud_(3,1,6) model builds upon the Cloud_(3,1) foundation by incorporating six additional VRE/SWIR bands, constituting a third modal input. This extension equips the model with a broader spectrum of information. The Cloud_(3,1,6,3) model further refines the architecture by incorporating three additional Ca/WV/Cir bands as a fourth modal input. This model encompasses data from all critical bands, aiming to further elevate the expressiveness and generalization capability of the model in multimodal fusion tasks through comprehensive spectral coverage.

**Figure 9:**
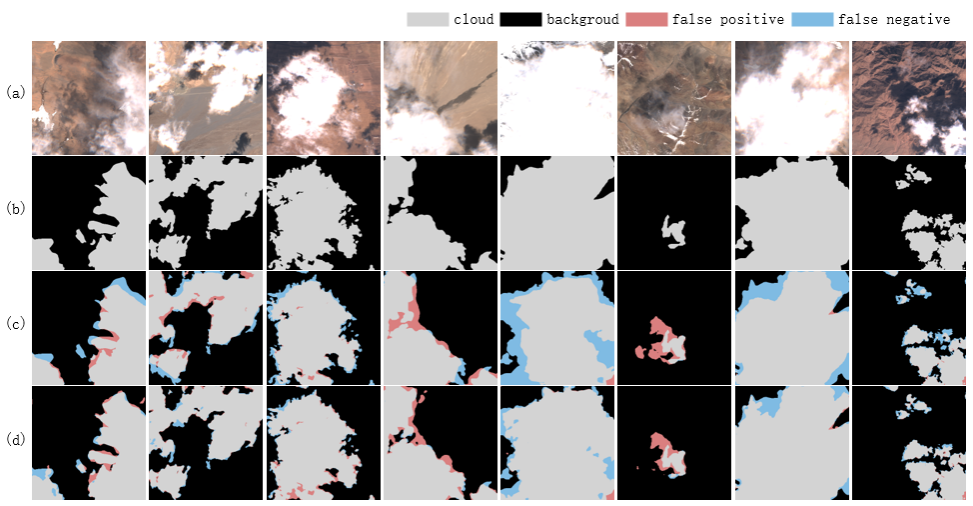
Important role of multimodal fusion module in cloud detection. (**a**) Image, (**b**) ground truth, (**c**) Cloud_4, and (**d**) Cloud_(3,1).

As shown in Table 3, compared with the single-modal models using multi-band direct concatenation, the models introducing multimodal fusion, and Cloud_(3,1,6,3)) exhibit significant superiority in various performance indicators. Notably, the Cloud_(3,1) model’s F1 score rises from 96.50% to 96.90% compared to the Cloud_4 model, and its MIoU increases from 93.38% to 94.09%. The improvement of the cloud detection performance by the multimodal fusion module can be intuitively reflected in Figure 9. This result indicates that treating visible light and near-infrared bands as independent modal inputs and fusing them can effectively mine and utilize the complementarity between them, thereby improving the model’s performance.

The Cloud_(3,1,6,3) model achieves the highest values in all evaluation indicators, which further confirms that multimodal fusion can make full use of richer spectral information, significantly enhancing the model’s expression and generalization abilities. In contrast, the direct stitching of multiple bands may fail to fully capture the potential relationships between modalities. Furthermore, the multimodal fusion model’s performance is particularly outstanding in terms of recall, which demonstrates that multimodal fusion can significantly improve the model’s detection ability for cloud targets by effectively reducing the missed detection rate. Even in more complex scenarios, as shown in Table 4, the Cloud_(3,1,6,3) model still achieves the highest values on all evaluation metrics.

**Contribution of the unified multimodal fusion module to the network performance.** We deploy the unified multimodal fusion module proposed in this paper on two classic segmentation models, UNet [56] and DeepLabV3+ [57], to evaluate its impact on model performance. In the baseline models, UNet and DeepLabV3+ directly concatenate the 13 spectral bands as input. In contrast, M_UNet and M_Deep perform the following: (1) divide the 13 bands into four modality groups based on their spectral characteristics, (2) process them through our unified multimodal fusion module for coordinated feature extraction and cross-modal fusion, then (3) feed the integrated features into the original network architectures with only input channel dimensionality adjustments. This efficient implementation preserves all subsequent layers while enabling effective multimodal processing as evidenced by the consistent performance improvements. As shown in Table 5, the integration of the multimodal fusion module results in consistent improvements in key evaluation metrics, for both UNet and DeepLabV3+. These improvements demonstrate the module’s ability to effectively integrate multimodal information, thereby enhancing the segmentation performance of the models.

For UNet, the improvement in precision is particularly significant, demonstrating the module’s capability to enhance the model’s ability to identify fine-grained details. For DeepLabV3+, although the improvement is relatively modest, the consistent performance gains across both models highlight the module’s adaptability to different architectures and its potential for handling multimodal data effectively.

**Figure 10:**
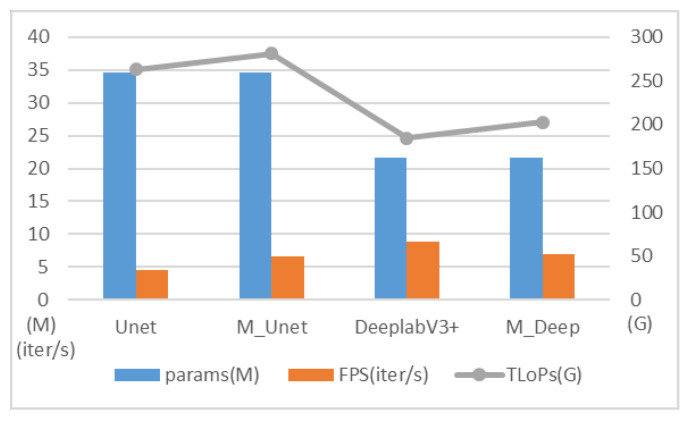
Comparison of the impact of unified multimodal fusion modules on network efficiency.

Figure 10 shows the comparison of the models in terms of efficiency. Compared with UNet, M_UNet exhibits a limited increase in both computational complexity and parameter count, yet achieves a significant improvement in FPS, indicating that the incorporation of the multimodal fusion module effectively optimizes the inference speed of the UNet architecture. UNet itself retains rich detailed information through its skip connection design, performing exceptionally well in tasks that demand high recall rates. The inclusion of the multimodal fusion module not only further enhances the feature extraction capabilities of M_UNet, improving both accuracy and MIoU, but also boosts the inference efficiency by efficiently integrating features and minimizing redundant calculations.

DeepLabV3+ achieves efficient feature extraction through the utilization of atrous convolution and ASPP modules, making it suitable for processing high-resolution images. The integration of the multimodal fusion module further enhances the feature integration capabilities of DeepLabV3+, leading to improvements in both the recall rate and MIoU. However, when compared to the lightweight design of DeepLabV3+, M_Deep introduces additional computational steps, resulting in a decrease in inference speed.

**Ablations study.** We establish a baseline using four modal data inputs (consisting of 13 bands) and the backbone network. Subsequently, we modify the networks to verify the contribution of each module as detailed in Table 6. Our conclusions are as follows: (1) The baseline model exclusively utilizes the backbone to process the 13 bands of multimodal data. Although it achieves high performance, there remains potential for enhancement in recall and MIoU, suggesting limitations in the model’s feature extraction and fusion capabilities. (2) The incorporation of the expert module (EM) into the baseline model leads to improvements in accuracy, precision, F1-score, and MIoU. These findings indicate that the EM effectively extracts features from multimodal data, thereby enhancing the model’s classification accuracy and segmentation performance. However, a slight decline in recall, from 96.33% to 96.26%, may be attributed to potential information loss during the feature extraction process facilitated by the EM. (3) Upon further integrating the Adaptive Fusion Module (AFM) with the EM-enhanced baseline model, all model metrics attain optimal values. Specifically, recall experiences a notable improvement, increasing from 96.26% to 96.58%. These results demonstrate that the AFM module effectively merges multimodal features, addressing the shortcomings of the EM module in detailed information extraction. Consequently, a superior balance between precision and recall is achieved, further augmenting the model’s overall performance.

Our model achieves competitive accuracy with 69.814 M parameters and 271.599 G FLOPs, requiring 291.336 MB memory. On a NVIDIA RTX 3090 GPU, it processes images at 4.58 FPS (218.4 ms per inference), making it suitable for offline batch processing (e.g., remote images analysis). Future work will focus on optimizing the model’s efficiency through hardware-software co-design, ensuring its viability for large-scale real-time applications.

In conclusion, the ablation experimental results emphasize the significant contribution of the proposed unified multimodal fusion module (EM + AFM) in improving model performance. The pivotal role of this unified module in the feature extraction and fusion of multispectral data is clearly validated.

### 4.4. Comparisons

To comprehensively evaluate the performance of the proposed model in cloud detection tasks, we utilized thirteen spectral bands from the WHUS2-CD dataset as input features for the network architecture. A rigorous comparative analysis was conducted against both classical semantic segmentation frameworks, namely UNet [56] and Deeplabv3+ [57], and state-of-the-art methodologies in the field, including AFMUnet [12], CSDFormer [39], and TransGA-Net [48]. It is noteworthy that these comparative models represent distinct architectural paradigms: AFMUnet is fundamentally based on the Convolutional Neural Network (CNN) framework, CSDFormer incorporates the Transformer architecture, and TransGA-Net adopts a hybrid CNN–Transformer approach, thereby providing a comprehensive benchmark for evaluating the proposed model’s performance across different architectural paradigms.

**Figure 11:**
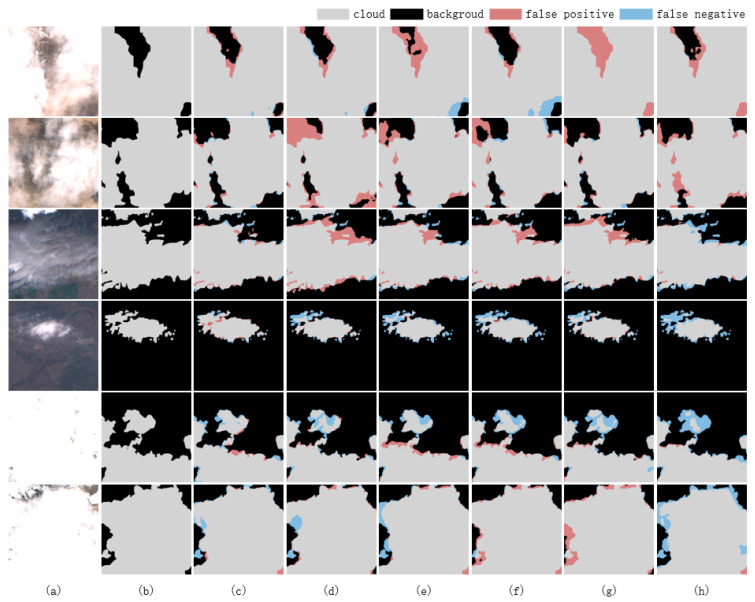
Comparison of cloud detection results using various methods under various land covers on the WHUS2-CD dataset. (**a**) Image, (**b**) ground truth, (**c**) ours, (**d**) Unet, (**e**) deeplabv3+, (**f**) AFMUnet, (**g**) CSDFormer, and (**h**) TransGA-Net.

In the context of cloud detection utilizing the WHUS2-CD dataset, the proposed model exhibits superior performance as evidenced by its overall accuracy of 98.91%, recall of 96.58%, F1-score of 96.99%, and MIoU of 94.26%, as detailed in Table 7. These metrics outperformed those of comparison models, thereby validating the efficacy of leveraging multispectral information for cloud detection tasks. This is shown in Figure 11.

**Figure 12:**
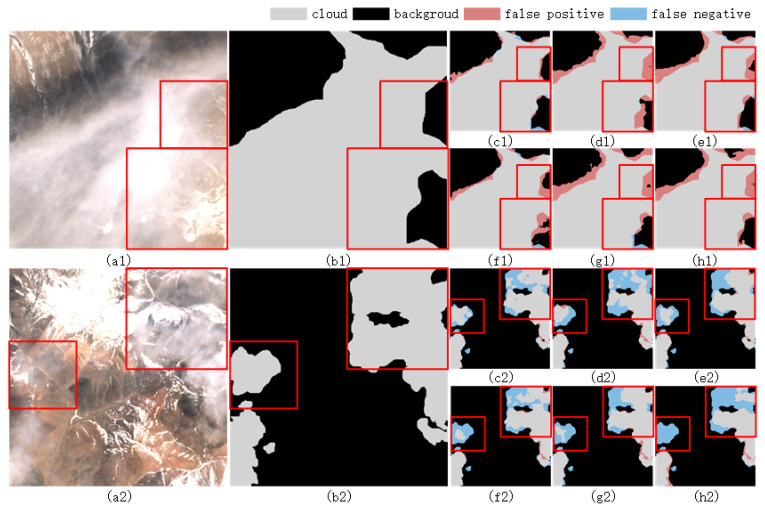
Comparison of cloud detection results using various methods in a typical complex scene with coexisting snow under thin clouds on the WHUS2-CD+ dataset. (**a1**,**a2**) Image, (**b1**,**b2**) ground truth, (**c1**,**c2**) ours, (**d1**,**d2**) Unet, (**e1**,**e2**) deeplabv3+, (**f1**,**f2**) AFMUnet, (**g1**,**g2**) CSDFormer, and (**h1**,**h2**) TransGA-Net.

To further challenge the model’s capabilities, the WHUS2-CD+ dataset was introduced, which augmented the original WHUS2-CD dataset with four additional images depicting snow/ice scenes. As illustrated in Table 8, the proposed model achieved an overall accuracy of 98.65%, surpassing that of UNet (97.95%), DeepLabV3+ (98.40%), AFMUnet (98.57%), CSDFormer (98.49%), and TransGA-Net (98.25%). This result underscores the model’s robustness in handling complex scenes, particularly in discriminating between clouds and snow/ice. Specifically, the proposed model demonstrated a precision of 97.42%, significantly higher than that of other models, suggesting a low false detection rate in distinguishing clouds from snow/ice. Although its recall rate of 93.96% was slightly lower than UNet’s 96.04%, the combination of high precision and recall indicated that the model maintained strong detection capabilities while minimizing false detections. Notably, the model’s F1-score of 95.61% was the highest among all compared models, further affirming its comprehensive performance in complex scenes. Additionally, its MIoU of 91.83% exceeded that of other models, indicating superior accuracy in pixel-level segmentation tasks. Figure 12 shows that in a typical complex scene with coexisting snow under thin clouds, the proposed model shows excellent detection ability and can effectively distinguish cloud from snow/ice, while performing well in detail retention and boundary clarity.

The proposed model’s superiority was consistent across models of varying architectural paradigms, suggesting that its design effectively integrates multi-spectral and multi-scale information, thereby enhancing cloud detection performance. These findings demonstrate that the model performs exceptionally well on both WHUS2-CD and WHUS2-CD+ datasets, particularly in complex scenes involving cloud and snow/ice discrimination. The exceptional performance of the proposed model can be attributed to its full utilization of multispectral information, adaptability to multiresolution data, and robustness in complex scenes. These attributes position the model as a promising candidate for a wide range of potential applications in remote sensing cloud detection tasks.

## 5. Discussion

**Advantage.** The unified multimodal fusion module introduced in this paper exhibits remarkable capabilities in processing multi-scale features, accommodating inputs of diverse resolutions, and enhancing the model’s generalization ability. It effectively integrates multimodal information, harnessing the spectral characteristics of various bands, particularly adept at distinguishing between cloud and snow/ice in complex scenes. Leveraging the strengths of multimodal data, the model surpasses other comparative models across various performance metrics and demonstrates substantial potential in practical applications, notably in differentiating intricate land cover types.

**Disadvantage.** The model’s performance depends heavily on input data quality, as noise or missing data can degrade detection accuracy. Additionally, processing multi-spectral, multi-resolution, and multimodal data increases computational complexity, especially for large-scale remote sensing tasks, requiring substantial resources. While the model excels in distinguishing clouds from snow/ice, challenges remain in extreme scenarios, such as thin cloud cover or highly similar spectral signatures, leading to potential false positives or missed detections. These limitations suggest key areas for future research: robust preprocessing for noisy data, computational efficiency optimization, enhanced edge-case detection through improved features or physical models, and expanded training datasets for better generalization.

## 6. Conclusions

In this paper, we propose an innovative multimodal data fusion method that aims to solve the challenge in network architecture design when dealing with a variable number of modalities. Unlike existing methods, the proposed method does not require structural modifications to the network architecture when introducing new modalities, thus significantly reducing the incremental computational cost and improving the overall efficiency. In addition, the proposed multimodal data fusion module possesses powerful generalization capabilities and can be seamlessly integrated into other network architectures in a plug-and-play manner, which greatly enhances the practicality and flexibility of the module.

Based on this method, we further construct a multimodal cloud detection model, M2Cloud. Through the deep integration of multimodal data, we achieve excellent performance on the public multimodal datasets WHUS2-CD and WHUS2-CD+, reaching or even surpassing the current state of the art (SOTA). The experimental results demonstrate that M2Cloud performs exceptionally well in the unified multimodal cloud detection task, which not only verifies its effectiveness but also offers a feasible reference method for the construction of similar models. The research in this paper provides new ideas and technical support for the field of multimodal data fusion and cloud detection, and holds important theoretical significance and application value.

However, real-world deployment may require additional optimization for resource-constrained edge devices—a direction we plan to explore in future work. While our current cosine-based identifiers work well for the sensor configurations in this study, extending them to novel sensor types requires further investigation. Important directions for future work include developing adaptive methods for new sensor types, incorporating atmospheric physical models to better handle edge cases, and employing physics-guided learning to enhance performance on difficult detection scenarios such as thin cloud layers. Emerging fusion paradigms also warrant investigation: graph-based approaches for the explicit modeling of spectral–spatial cloud relationships, and multimodal transformers for cross-sensor feature attention. These may particularly benefit edge-case detection.

## Figures and Tables

**Table 1:** Information of the datasets.

Datasets	Satellite	Train	Test	Size	Resolution	Bands
WHUS2-CD+	Sentinel-2	24	12	10,980 × 10,980 3660 × 3660 1830 × 1830	10 m 20 m 60 m	Blue, Green, Red, Near InfraRed 5, 6, 7, 8A, 11 (SWIR), 12 (SWIR) 1, 9, 10
CloudSEN12+	Sentinel-2	8490 687	975 85	509 × 509 2000 × 2000	10 m	Blue, Green, Red, Near InfraRed 5, 6, 7, 8A, 11 (SWIR), 12 (SWIR), 1, 9, 10, TOA
GF-1 WHU	Gaofen1(WFV)	108		16,633 × 15,425	16 m	Blue, Green, Red, Near InfraRed
MODIS	MODIS	1192	150	512 × 512	500 m 1000 m	Blue, Green, Red 18, 20, 23, 28, 29, 31, 32
95-Cloud	Landsat 8	75	20	384 × 384	30 m	Blue, Green, Red, Near InfraRed
Levir_CS	Gaofen1 (WFV)	3068	1100	1200 × 1340	16 m	Blue, Green, Red, Near InfraRed DEM (Digital Elevation Model)

**Table 2:** Comparison of evaluation metrics of different baseline models on the WHUS2-CD dataset.

Model	Accuracy	Precision	Recall	F1-Score	MIoU
Cloud_3	98.59%	96.67%	95.53%	96.01%	92.65%
Cloud_4	98.74%	97.19%	95.83%	96.50%	93.38%
Cloud_10	98.82%	97.66%	95.81%	96.71%	93.75%
Cloud_13	98.83%	97.33%	96.18%	96.75%	93.82%

**Table 3:** The evaluation metrics of different multimodal models on the WHUS2-CD dataset.

Model	Accuracy	Precision	Recall	F1-Score	MIoU
Cloud_4	98.74%	97.19%	95.83%	96.50%	93.38%
Cloud_(3,1)	98.88%	97.51%	96.30%	96.90%	94.09%
Cloud_10	98.82%	97.66%	95.81%	96.71%	93.75%
Cloud_(3,1,6)	98.89%	97.54%	96.32%	96.92%	94.13%
Cloud_13	98.83%	97.33%	96.18%	96.75%	93.82%
Cloud_(3,1,6,3)	98.91%	97.41%	96.58%	96.99%	94.26%

**Table 4:** The evaluation metrics of different multimodal models on the WHUS2-CD+ dataset.

Model	Accuracy	Precision	Recall	F1-Score	MIoU
Cloud_(3,1)	98.45%	96.04%	94.05%	95.02%	90.81%
Cloud_(3,1,6)	98.50%	97.60%	92.83%	95.01%	90.87%
Cloud_(3,1,6,3)	98.65%	97.42%	93.96%	95.61%	91.83%

**Table 5:** Comparison of evaluation metrics of different multimodal models on the WHUS2-CD dataset.

Model	Accuracy	Precision	Recall	F1-Score	MIoU
UNet	98.78%	96.55%	96.77%	96.66%	93.66%
M_UNet	98.82%	97.52%	95.96%	96.73%	93.78%
DeepLabV3+	98.71%	97.93%	94.88%	96.34%	93.10%
M_Deep	98.77%	97.66%	95.51%	96.56%	93.48%

**Table 6:** Evaluation of the proposed unified multimodal fusion module with different settings on the WHUS2-CD dataset.

EM	AFM	Backbone	Accuracy	Precision	Recall	F1-Score	MIoU
		✓	98.83%	97.21%	96.33%	96.76%	93.85%
✓		✓	98.86%	97.46%	96.26%	96.85%	94.01%
✓	✓	✓	98.91%	97.41%	96.58%	96.99%	94.26%

**Table 7:** The evaluation metrics of different models on the WHUS2-CD dataset.

Model	Accuracy	Precision	Recall	F1-Score	MIoU
UNet	98.78%	96.55%	**96.77%**	96.66%	93.66%
DeepLabV3+	98.71%	**97.93%**	94.88%	96.34%	93.10%
AFMUnet	98.75%	97.88%	95.19%	96.48%	93.35%
CSDFormer	98.80%	97.21%	96.13%	96.68%	93.71%
TransGA	98.80%	97.71%	96.25%	96.67%	93.69%
ours	**98.91%**	97.41%	96.58%	**96.99%**	**94.26%**

**Table 8:** The evaluation metrics of different models on the WHUS2-CD+ dataset.

Model	Accuracy	Precision	Recall	F1-Score	MIoU
UNet	97.95%	91.92%	**96.04%**	93.86%	88.84%
DeepLabV3+	98.40%	95.52%	94.29%	94.90%	90.59%
AFMUnet	98.57%	96.08%	94.82%	95.44%	91.53%
CSDFormer	98.49%	95.55%	94.87%	95.21%	91.13%
TransGA	98.25%	96.05%	92.71%	94.30%	89.60%
ours	**98.65%**	**97.42%**	93.96%	**95.61%**	**91.83%**

## Data Availability

The raw data supporting the conclusions of this article will be made available by the authors on request.
